# Validation and Comparison of a Model of the Effect of Sea-Level Rise on Coastal Wetlands

**DOI:** 10.1038/s41598-018-19695-2

**Published:** 2018-01-22

**Authors:** Laura A. Mogensen, Kerrylee Rogers

**Affiliations:** School of Earth and Environmental Science, University of Wollongong, Wollongong, NSW 2522 Australia

## Abstract

Models are used to project coastal wetland distribution under future sea-level rise scenarios to assist decision-making. Model validation and comparison was used to investigate error and uncertainty in the Sea Level Affecting Marshes Model, a readily available model with minimal validation, particularly for wetlands beyond North America. Accurate parameterisation is required to improve the performance of the model, and indeed any spatial model. Consideration of tidal attenuation further enhances model performance, particularly for coastal wetlands located within estuaries along wave-dominated coastlines. The model does not simulate vegetation changes that are known to occur, particularly when sedimentation exceeds rates of sea-level rise resulting in shoreline progradation. Model performance was reasonable over decadal timescales, decreasing as the time-scale of retrospection increased due to compounding of errors. Comparison with other deterministic models showed reasonable agreement by 2100. However, given the uncertainty of the future and the unpredictable nature of coastal wetlands, it is difficult to ascertain which model could be realistic enough to meet its intended purpose. Model validation and comparison are useful for assessing model efficacy and parameterisation, and should be applied before application of any spatially explicit model of coastal wetland response to sea-level rise.

## Introduction

Observed and predicted increasing rates of sea-level rise (SLR) have caused considerable concern for the long-term sustainability of coastal wetlands around the world^1,2^. Saline coastal wetlands occur in low energy, saline environments and lie within narrow elevation ranges associated with tidal inundation. These environments are essential to the livelihood of many societies around the world and provide a range of critical regulating services, many of which are of considerable economic value to society, including shoreline armouring against storms and erosion, nutrient cycling and carbon sequestration^3^. However, due to their characteristic position within the intertidal zone, these areas are considered to be one of the most vulnerable to SLR. As coastal wetlands are inextricably linked to sea level and tidal inundation, changes in tidal regimes of coastal environments influenced by SLR during the twenty-first century will likely see a significant influence on the distribution of coastal wetlands and, in turn, the valuable services they provide. In order to effectively plan for such situations and successfully manage these coastal environments, models of the response of coastal wetlands to SLR have been developed, often in advance of adequate understanding of the spatial and temporal impacts of SLR at the local and regional scale.

Modelling impacts of SLR has now moved beyond using simple ‘bathtub’ approaches, whereby coastal landscapes are flooded at rates corresponding to sea-level rise, and now recognise a range of processes that influence coastal landscape geomorphology^4,5^. The basis for most models of the response of coastal wetlands to SLR is that persistence of these environments is dependent upon a variety of interconnected processes that build the wetland surface, such as sedimentation, and those that decrease the relative surface elevation, such as soil compaction or inundation resulting from rising sea levels. Increases in frequency and duration of inundation caused by SLR disrupt the balanced environment of wetlands, leading to increases in sediment deposition and maintenance of wetland position, landward shifts in vegetation distribution or, in cases of extreme disequilibrium between wetland surface gains and rates of relative SLR, complete submergence of the coastal wetland^6,7^. Many of the current numerical models are concerned with above-ground depositional processes that successively increase elevation over time without providing due consideration of erosional processes on changes in wetland surface elevation^8,9^. Other models have explicitly considered erosional and depositional processes^10–12^ and do not explicitly incorporate below-ground processes. Some models focus on the ecological influence of root and organic matter additions to substrates and associated effects on wetland surface elevation^13,14^. Most models are particularly sensitive to substrate elevation^15^, and high resolution digital elevation models (DEMs), typically derived from light detection and ranging techniques (Lidar), are useful input datasets for spatially explicit models^5,15,16^.

In theory, the greater complexity of models allows for a closer approximation of the wetland surface elevation responses to SLR. However, the superiority of more complex models has been questioned, with many suggesting that the greater parameterisation allows for increased potential of additional errors and uncertainty^17,18^. Models of low complexity, however, may also contain much error as a result of structural uncertainty. It is, therefore, debated which type of model is most relevant and useful in modelling of complex environmental systems^18,19^. The cumulative effect of this philosophical debate and demand for spatial projections of the response of coastal wetlands to SLR to assist with coastal adaptation planning is increasing in the use of readily available models that do not require conceptualisation and can be used with minimal parameterisation. In particular, the Sea Level Affecting Marshes Model (SLAMM), which was initially conceptualised in the 1970s and calibrated using empirical knowledge of the response of wetlands associated with the North Atlantic Ocean, is increasingly being used to project the spatial distribution of wetlands under future sea-level scenarios in a range of environmental settings^20–22^. However, concerns regarding the application of models to systems which display distinctly different geomorphological, hydrological and ecological characteristics to the wetland environment in which the model was initially conceptualised and validated are arising^23,24^. These concerns emphasise the need to validate models developed elsewhere prior to application to a different environmental setting. In spite of this, readily available models are being applied to environmental settings for which they have not been conceptualised, calibrated against and for which there is minimal empirical data or inadequate validation.

SLAMM is one of the most widely used spatial landscape models (described in Supplementary Material 1). It is a complex, non-hydrodynamic model that simulates six primary processes affecting the survival of coastal wetlands with long term SLR including inundation, erosion, overwash, saturation, salinity and accretion, of which inundation and accretion are most frequently implemented. The ability of SLAMM to adequately describe the response of wetlands to SLR has been called into question, and numerous revisions of SLAMM have occurred as a consequence (e.g. Discussions regarding version V^25–27^). Few attempts have been made at model validation and calibration, particularly in regions beyond the wetlands associated with the North Atlantic Ocean^15^. This ultimately affects the understanding of the unique ecosystems around the world and the management of these vulnerable environments. For example, in Australia SLAMM has been applied to wetlands of northeastern New South Wales, and while it has been acknowledged that model outputs could be unrealistic due to the differences between North American and Australian wetlands, SLAMM generated data was still used to provide insight into the possible impacts of SLR^28^. Further studies on the impacts of SLR on coastal ecosystems of southeastern Queensland provided confident conclusions from SLAMM without prior assessment of the reliability of the model to adequately describe the system^22,29,30^. Validation of SLAMM is, thus, overdue for coastal regions beyond North America, and analyses from southeastern Australia are relevant to other high energy wave-dominated coastlines globally where coastal wetlands are located in sheltered positions behind a coastal barrier.

Retrospective validation of SLAMM, a type of predictive analysis, has been undertaken for validation purposes^20,31^. Coding within SLAMM prevents backward time-steps, and retrospective validation has been undertaken using other approaches. Ideally retrospective validation would be undertaken using a historic DEM from which SLAMM can project prior vegetation changes for comparison with contemporary vegetation distribution (i.e. historic high resolution Lidar-derived digital elevation model). We are unaware of any validations of SLAMM using a historic DEM as Lidar technology is relatively new and historic elevation data is generally regarded to be of inadequate accuracy and resolution as input data within SLAMM^27^. In the absence of historical elevation information, an elevation surface can be produced within SLAMM using the vegetation preprocessor, whereby the maximum and minimum elevation of a given vegetation class is determined and assigned to the upper and lower boundaries of the mapped class with elevations between these boundaries being linearly interpolated. The resulting elevation surface, a continuous layer for the study site derived from vegetation class boundaries, is then utilised for subsequent projections. Alternatively, retrospective analysis has been achieved by forcing a contemporaneous DEM to be representative of a prior time period by adjusting elevations based on the rate of relative SLR over the period that retrospective analysis occurred^20,31^. In effect, this approach applies a different backward stepping model and projects SLAMM forward; as the models are different, it is not surprising that differences in the SLAMM projection and contemporary vegetation distribution occurs. Retrospective validation achieved by forcing a DEM backwards and using this as the basis for model projections has found ‘reasonable’ agreement between current vegetation distribution and projected vegetation distribution, but this is anticipated given the relative short periods of retrospection (~30 years for both analyses), and the modest rates of SLR over this period. This approach to retrospective validation was undertaken with model output being compared to vegetation distribution over only a decadal period of retrospection^32,33^.

Model comparison^33–35^ also provides useful insights into the structure and function of SLAMM. SLAMM outputs have been compared to similar projection scenarios applied to a regionally developed model (saltmarsh model for the Yangtze estuary, or SMM-YE)^35^, with poor agreement found in the distribution of saltmarsh at 2100, particularly under the high SLR scenario. Using a synthetic elevation surface, as opposed to a Lidar derived DEM, reasonable agreement was found between SLAMM and the Marsh Accretion and Inundation Model (MAIM)^34^, however parameterisation of SLAMM, the SLR scenario and period of projection was not indicated. Comparison with a range of neutral models over a two-year period of retrospection found more accurate simulation of wetland change using SLAMM, while aggregation of vegetation classes expectedly improved performance of both SLAMM and neutral models^33^.

This study aims to validate SLAMM and compare SLAMM outputs to other model outputs to assess its suitability for application in a wave-dominated estuary of southeastern Australia, a setting which has received little calibration or validation. Coastal wetlands in this region are primarily located landward of sand barriers that form at the entrance of most estuaries (few wetlands are associated with sheltered shorelines of drowned river valleys such as Sydney Harbour, Port Hacking and Hawkesbury River)^36^. This creates conditions where tides can be either amplified or attenuated as they ebb and flow along estuaries and across the intertidal zone^37,38^, and which has an important, yet highly variable influence on wetland distribution. This coastline also supports a complex mosaic of wetland communities, that include mangrove forests of *Avicennia marina* and *Aegiceras corniculatum* where inundation frequency is relatively high (i.e. <50%), shrub and dwarf mangrove where inundation frequency is moderate (i.e. 50–25%), mixed species saltmarsh communities when inundation frequency is low (i.e. <25%), and landward forests of *Casuarina glauca* or *Melaleuca* species. The nomenclature and distribution of these zones does not correspond to existing National Wetland Inventory^39^ vegetation classes used in SLAMM and requires calibration to ensure classes respond appropriately. Following model set-up, retrospective validation using SLAMM simulations between 1949–1997, and 1986–1997; and comparison of SLAMM outputs with other model outputs was undertaken. Models used in model comparison included SLAMM, a temporally adjusted version of a spatially-explicit accretion model^40^, henceforth termed comparison model 1 (or CM1), and an empirically-derived wetland elevation model developed for the study site using empirical data from the study site^41^, henceforth termed comparison model 2 (or CM2). In doing so, this study demonstrates the need for model calibration for regionally specific conditions, and model validation to assist with interpretation of model efficacy, and provides a framework for model validation and comparison prior to interpretation of model projections.

## Results

### Model setup

Data calibration and evaluation was undertaken to ensure that input data sets corresponded to conditions at the study site. This study focused on three input parameters: DEM, vegetation classes and tidal attenuation. These parameters were calibrated to correspond to empirical data from the study site. Methods and results for DEM calibration and vegetation calibration are provided in Supplementary Material 2. Modelling and evaluation of tidal attenuation to define the tidal range parameter within SLAMM is provided below.

**Figure 1:**
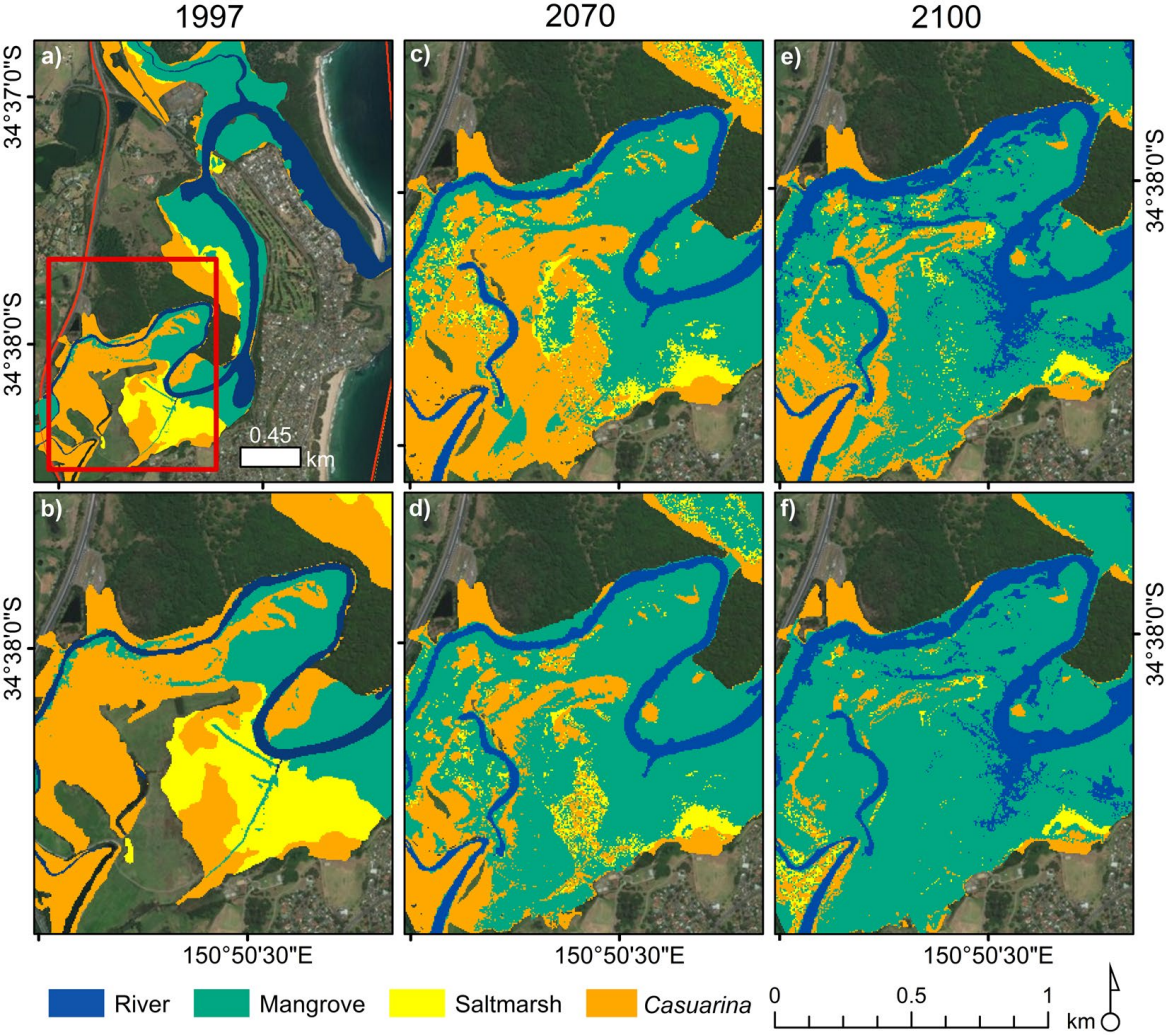
Different model outputs when using a basic method to account for tidal attenuation. (**a**) Minnamurra River with inset indicating focus tidal attenuation site (**b**) The initial 1997 vegetation distribution in the focus tidal attenuation study site (**c**) Vegetation distributions at 2070 when tidal attenuation is included in modelling (**d**) Vegetation distributions simulated for 2070 when a single tidal range is used for the entire estuary (**e**) Model output for 2100 when tidal attenuation is accounted for within the model (**f**) Modelled vegetation distributions at 2100 when tidal attenuation is not considered in the modelling process. (Imagery Source: Esri, DigitalGlobe, GeoEye, Earthstar Geographics, CNES/Airbus DS, USDA, USGS, AeroGRID, IGN, and the GIS User Community. Figure generated using ArcMap Version 10.4.1).

Accounting for tidal attenuation within the modelling process resulted in distinct differences in modelled vegetation distributions by 2100 when compared to the model output produced when using a single value of tidal range for the entire study site. Differences were most notable in the upper reaches of the tidally influenced portion of Minnamurra River (Fig. 1). By vegetation class, greatest differences were apparent within mangrove and *Casuarina*, whilst saltmarsh extents modelled to remain by the year 2100 were relatively similar when accounting for tidal attenuation and when applying a global tidal range value. Proliferation of mangrove was both steadier and to a lesser extent when tidal attenuation was included in the model, with mangrove extent at the final year of modelling (29.13 ha) being more than half that simulated when tidal attenuation was not included in modelling (63.89 ha). In contrast, a greater area of *Casuarina* and saltmarsh was simulated over the entire modelling period (1997–2100) when accounting for tidal attenuation.

### Retrospective validation

**Figure 2:**
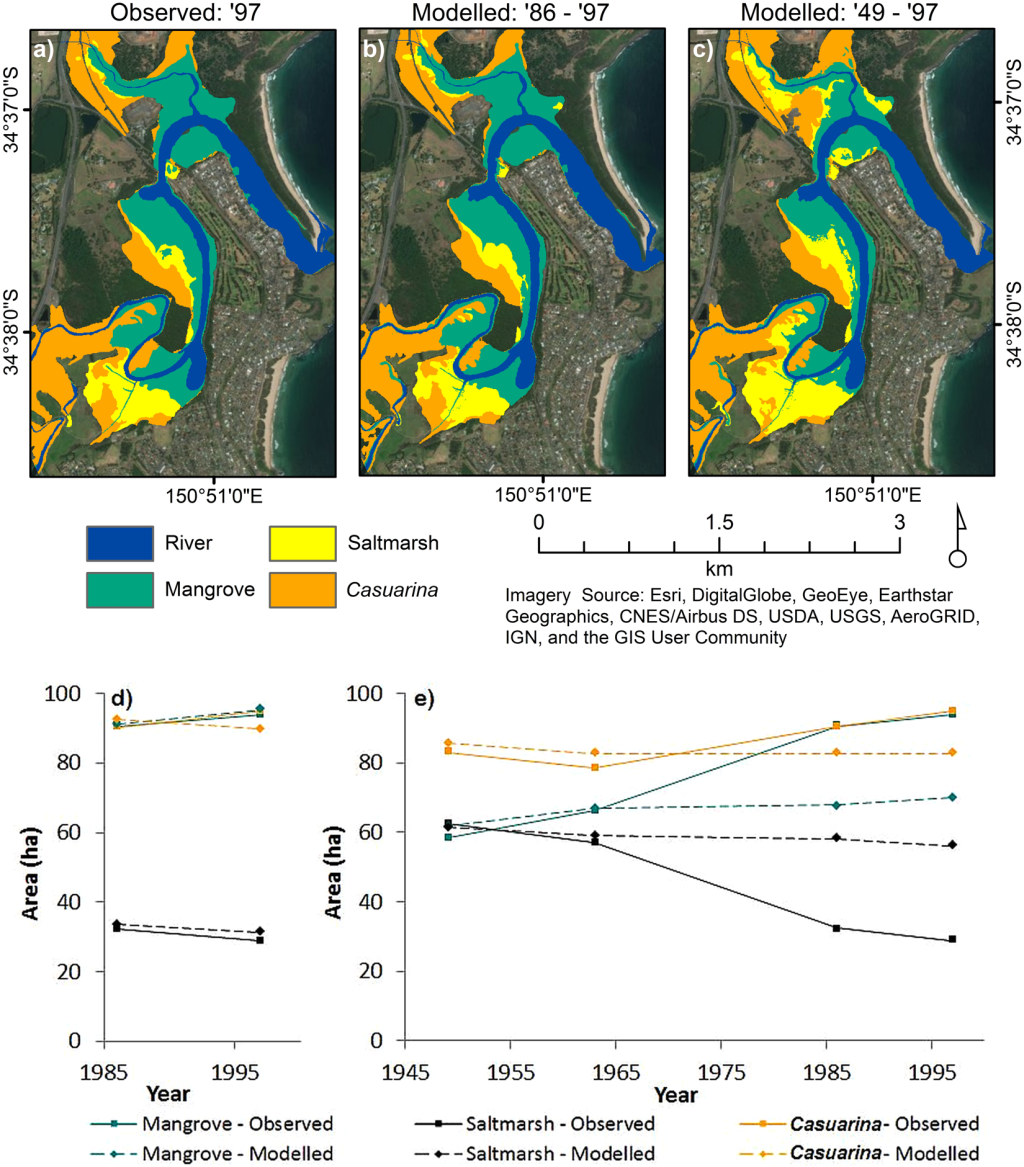
Retrospective validation of SLAMM over two time periods 1986–1997 and 1949–1997. Modelled vegetation distribution at 1997 based on retrospective analyses from (**a**) 1986 to 1997, and (**b**) 1949 to 1997; (**c**) observed vegetation distribution at 1997 provided for reference. Observed (solid line) and modelled (dashed line) vegetation extent from (**d**) 1986 to 1997 and (**e**) 1949 to 1997. (Imagery Source: Esri, DigitalGlobe, GeoEye, Earthstar Geographics, CNES/Airbus DS, USDA, USGS, AeroGRID, IGN, and the GIS User Community. Figure generated using ArcMap Version 10.4.1 and Microsoft Excel 2010).

Modelled vegetation distribution for the period 1986–1997 was relatively consistent with those mapped by Chafer (1998), with predicted areal extents being within 10% of observed distributions for all classes. Of the wetland vegetation classes modelled, saltmarsh areas produced the least fit with the 1997 observed vegetation data (percentage area difference = 8.5%), whilst mangrove distributions were relatively well predicted (percentage area difference = 1.7%). Spatial differences between the mapped vegetation distribution and model outputs of vegetation distribution were evident. Mangrove zones upstream appeared to be overestimated by SLAMM, especially along the banks of the river, whilst further downstream mangrove distribution was considerably less than that mapped by Chafer (1998) (i.e. within the model comparison study site; Fig. 2a). In place of the mangrove zones, saltmarsh had erroneously been modelled to occur, contributing to the overestimation of this wetland class. A larger decrease in the distribution of saltmarsh was noted in the observed data than in that modelled using SLAMM.

Over a longer time period (1949–1997), greater differences were found between mapped and modelled vegetation distributions. Within this period, modelled distribution at 1963 displayed a relatively good fit to mapped distribution (Fig. 2e) and variance was within 10% for all vegetation classes, particularly the mangrove vegetation class (0.71%). However, significant deviations were evident between the mapped and modelled distributions by 1997 (Fig. 2b), with comparisons of mapped and modelled areas indicating an overall underestimation of mangrove (−25%) and *Casuarina* (−13%) areas and overestimation of saltmarsh (94%). More than a third of simulated saltmarsh areas (35%) were located within observed mangrove zones and only 47% of modelled saltmarsh distribution corresponded to the mapped saltmarsh distribution. Errors associated with *Casuarina* zones increased over time due to the model simulating a loss in the vegetation type over each temporal period as opposed to a mapped increase over the same period.

### Model comparison

**Figure 3:**
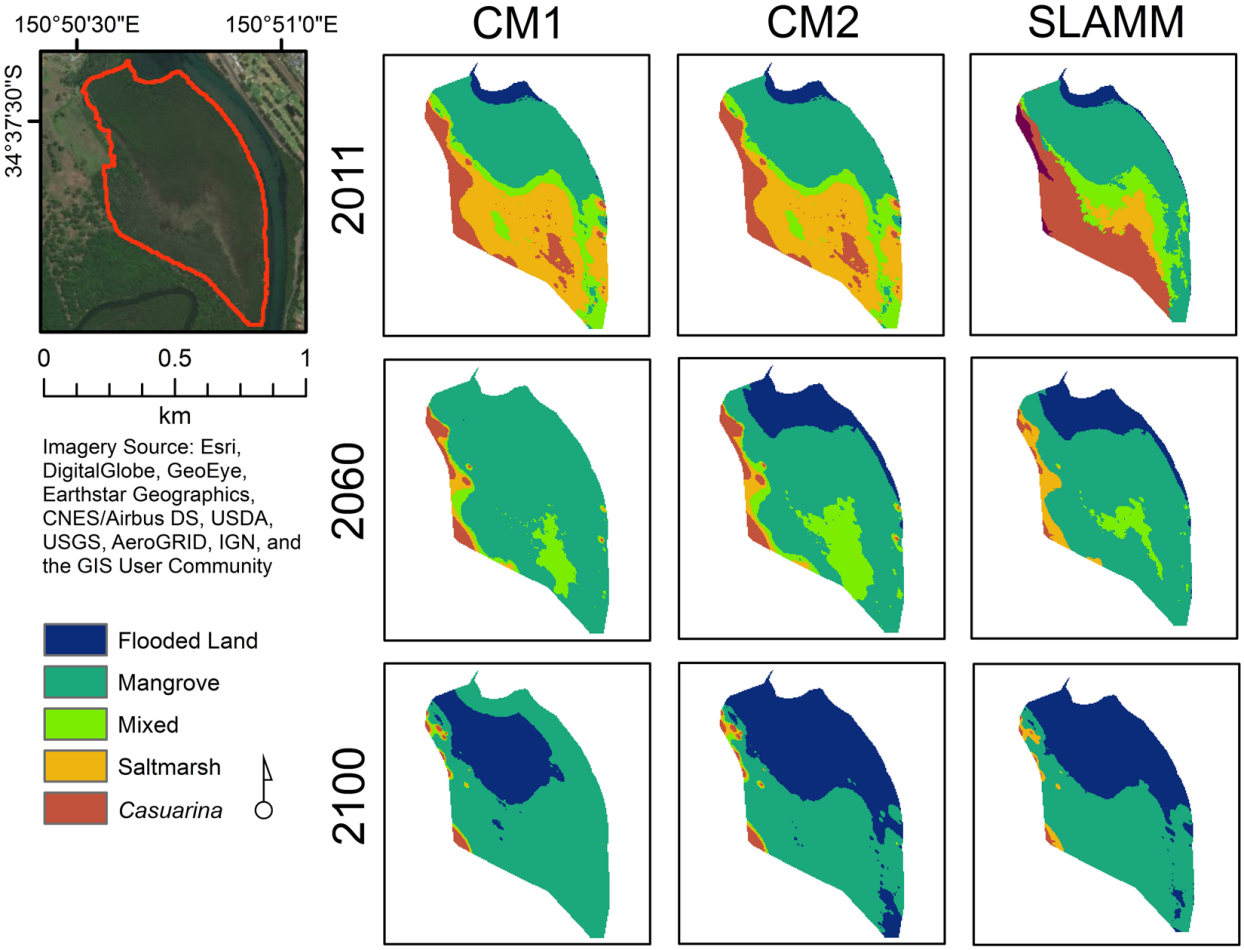
Model comparison of SLAMM, CM1 and CM2 at 2011, 2060 and 2100. Model simulations undertaken using 10 year time-steps and based on an A1FI (95%CI) SLR scenario only. (Imagery Source: Esri, DigitalGlobe, GeoEye, Earthstar Geographics, CNES/Airbus DS, USDA, USGS, AeroGRID, IGN, and the GIS User Community. Figure generated using ArcMap Version 10.4.1).

Differences in the initial vegetation distribution at 2011 (Fig. 3) was a result of the mode in which vegetation was defined within models, with SLAMM utilising a vegetation map as the base vegetation information, whilst CM1 and CM2 simulated initial wetland distributions based on vegetation-specific elevation ranges. This was particularly evident for saltmarsh and *Casuarina* areas.

Despite differences in initial vegetation distribution (see Methods for description of why initial vegetation distributions differed), the modelled vegetation distribution at 2100 for SLAMM and CM2 was relatively similar, with both models simulating a loss of mangrove to a final areal extent of 17.2 ha and 19.8 ha respectively. This primarily relates to similarities in the underlying mathematics conceptualised in both SLAMM and CM2. In contrast, CM1 was conceptualised on the basis of an exponential model of sediment accumulation with elevation, and consequently projected a moderate increase in mangrove area to cover an extent of 27.1 ha by the year 2100.

Differences likely resulting from the underlying mathematics of the models implemented were also evident in the simulation of wetland flooding and conversion to open water over the period 2011–2100. The spatial position of flooded areas predicted using SLAMM and CM2 were relatively similar, with changes from mangrove to inundated land initiating at the far northeastern border of the model comparison study site around 2060 and spreading throughout the area in a southerly direction (Fig. 3). By contrast, no flooded areas were simulated by CM2 until the year 2080, with significant proliferation of mangrove areas occurring prior to that time. Furthermore, initial conversion of mangrove to flooded land was not observed in the CM1 output from the northeastern boundary but rather at a point approximately 100 m from the river. Again, this likely relates to the exponential mathematics upon which CM1 is based, which resulted in the simulation of a mangrove stand on average 40 m wide at 2100 that behaves like a levee behind which flooding occurs.

Though the spatial pattern of change was variable between models, all three models predicted between a 95% and 100% loss of the saltmarsh zone by the end of the model simulation. Similar to saltmarsh zones, *Casuarina* and mixed zones displayed significant variation between models at the year 2011 yet the areal extent of these classes effectively converged by the year 2100.

### Sensitivity analysis

SLR, tidal range, salt boundary elevation, NAVD88-MTL and historic sea-level trend were the most sensitive factors within SLAMM (Table 1). Accretion rates were also calculated to be sensitive parameters regardless of the manner in which they were defined. In decreasing order of sensitivity, mixed, saltmarsh and mudflat classes were most sensitive to 10% variation in SLR, historic sea-level trend and NAVD88-MTL, while *Casuarina* and mangrove were most sensitive to SLR, tidal range and salt boundary elevation. Overall sensitivity of the model to SLR, great diurnal range and salt elevation boundary parameters indicated that the definition and magnitude of inundation were the most important factors affecting the model output, reflecting model conceptualisation. Errors in the characterisation of these parameters would, therefore, cause possibly significant inaccuracies in model output.

**Table 1:** Sensitivity analysis results indicating percentage change in each vegetation type following 10% variation in each parameter implemented within SLAMM. All values reported are percentages, where 0 indicates no effect occurs with variation in the parameter, positive values indicating a gain in vegetation areas and negative values indicating a loss. SLR was consistently calculated to have a significant effect on model output, with 10% variation in the parameter resulting in significant losses or growth of certain vegetation zones.

Parameter	Historic sea level trend	NAVD88 - MTL	Salt Boundary Elevation	Mangrove Accretion	GT Great Diurnal Tide Range	SLR by 2100
Casuarina	72	42	−97	0	243	287
Mangrove	9	5	7	−19	−145	28
Mudflat	−117	−69	−1	46	0	−430
Mixed	299	175	0	0	369	1228
Saltmarsh	118	71	0	0	313	448
Minimum	−117	−69	−97	−19	−145	−430
Maximum	299	175	11	46	369	1228
Mean	64	38	−13	4	130	262
Standard deviation	140	82	41	22	206	560

## Discussion

With projections of accelerated SLR for the twenty first century, there is increasing concern regarding the long-term sustainability of coastal wetlands. Managers are tasked with the responsibility of planning for future scenarios, responding to the potential vulnerability of the important coastal environments. Models provide opportunities to explore future scenarios under varying scenarios of SLR and thereby become an important instrument in a manager’s toolkit. However, the validity of a model used to support decision-making is crucial for a plausible and more reliable representation of the future system to be obtained. The validation and comparison approaches used in this study were more comprehensive than previous attempts that included some data calibration^33,35^ or short time-scale retrospective analyses^31,33^. The approach provides a useful framework for model validation and assessment, and we advocate such an approach prior to application of SLAMM, or indeed any model projecting wetland vegetation changes in response to SLR. Simulations of the wetland response to SLR were plausible, however certain conceptual limitations, and error and uncertainty, were associated with the application of SLAMM to the study site and extrapolation of relationships over long time scales. The results of this study are relevant to barrier estuaries of southeastern Australia and other coastal wetlands situated along wave-dominated coastlines. Errors and uncertainty specifically relate to potential problems with conceptualisation of vegetation change, inadequate treatment of surface elevation change (SEC) and insufficient consideration of the influence of site-specific geomorphology on tidal planes.

In all instances where retrospective analysis has been undertaken over short-timescales, including this study, reasonable agreement has been established between model projections and prior vegetation distributions derived from mapping of aerial photography^33,35^. In this study, the difference in extent between mapped and modelled distribution of vegetation classes in the period 1986–1997 ranged between 3.3% for mangrove and 9.4% for saltmarsh. For retrospective analysis over a period exceeding 30 years, the first time for which this has occurred, differences in mapped and modelled vegetation distribution were greater in the 1949–1997 retrospective analysis compared to the shorter period of retrospection. Greatest errors for each validation run, 1986–1997 and 1949–1997, were consistently associated with overestimated saltmarsh vegetation, and correspondingly underestimated mangrove extents. Increased errors over time associated with *Casuarina* zones appeared to be primarily related to an inverse pattern of change between the mapped and modelled distribution. That is, where *Casuarina* zones were mapped to increase over each temporal period, the model simulated a loss in the vegetation type over the same period. Spatial distributions of vegetation-specific inaccuracies were relatively similar within the model output of each validation run. The magnitude of these errors, however, varied according to the temporal period simulated.

Whilst errors in model output are indicative of SLAMM’s performance for the study site and southeast Australian coastal wetlands in general, the model output can only be as reliable as the input information used to define SLAMM. That is, error and uncertainty may be introduced to the model via both data and techniques employed to conduct the retrospective analysis. The technique employed in this study, and others, to produce elevation information for retrospective analyses effectively involves the application of a simple bath-tub model to a Lidar-derived DEM to back-step the input elevation model, and subsequent implementation of SLAMM for forward projections. As these models are different, it is not surprising that differences emerge between the mapped and modelled vegetation distributions and that these differences increase with the period of retrospection. A more effective approach for retrospective analysis, which would not require a prior high resolution DEM or application of bathtub techniques to back-step the DEM, would involve reverse time-steps of SLAMM simulations, with vegetation distribution being modelled on a simulated DEM, and comparison with prior vegetation distribution based on mapping from historic aerial or satellite imagery.

Irrespective of issues identified during retrospective analyses, through model set-up, tidal attenuation analysis and sensitivity analysis it was evident that careful treatment of input datasets was essential for modelling purposes. Here we have especially focussed on the effect of estuary geomorphology, specifically changing water depth and channel geometry^37^, on variation in tidal level within an estuary. This is pertinent as tidal levels demarcate areas of inundation, and therefore are inextricably linked to vegetation distribution^42^ and patterns of sedimentation and substrate elevation. This was demonstrated by partitioning discrete tidal values to subsites that were delineated in SLAMM. This basic method of accounting for tidal attenuation resulted in considerably different and, conceptually, more plausible vegetation distributions when compared to the model output produced when no tidal attenuation was considered. The inclusion of this process in the model is particularly important for barrier estuaries along wave-dominated coastlines where tide and wave energy are severely attenuated due to constriction of the tidal prism at estuary entrances^37^. Further improvements of SLAMM could be achieved by incorporating a modelled tidal plane surface that accounts for the effect of estuary geomorphology on both tidal attenuation and amplification.

Elevation data is the basis of most spatial modelling of the effect of SLR on coastal wetlands^15^. Model set-up and sensitivity analysis emphasised the importance of generating the most accurate DEM possible for modelling purposes, a fact that has been demonstrated many times^43,44^, yet is not prioritised in many applications of SLAMM and other spatial modelling exercises. This is especially true in modelling of low-lying wetlands where small errors in the surface representation result in large variations in the hydrological properties, geomorphic adjustments and simulation of wetland persistence or demise. Given the necessity of accurate elevation information, this study strongly advocates the expert processing of as-received Lidar data and considered derivation of the DEM in order to obtain an elevation surface that best represents the initial conditions of the system to be modelled. Furthermore, given the inherent uncertainty of spatial data, an accuracy assessment of a DEM generated is required so as to obtain an understanding of potential error and uncertainty in subsequent modelling efforts.

Treatment of accretion and SEC within SLAMM, while undergoing considerable refinement, requires careful calibration to ensure that parameterisation corresponds to site specific empirical data and that the potential errors in model output are reduced. The effect of subsurface processes of sediment compaction and consolidation, basement subsidence, and biotic processes of organic matter additions and decomposition on wetland substrate elevations is well established^45,46^, yet accretion and SEC are conceptualised as being synonymous in SLAMM. By using rates of SEC from SET-derived time-series data this problem was ameliorated. The essential need for high resolution site-specific empirical data for SLAMM parameterisation is pertinent for model output accuracy.

The relatively simple application of constant accretion rates within SLAMM has previously been identified as inadequate^27^, and subsequent inclusion of the accretion module in version 6 has attempted to account for spatial variation in accretion rates and ecogeomorphic feedbacks within wetlands that contribute to the persistence of wetlands over time^9,47^. To account for ecogeomorphic feedbacks, a modified version of the Marsh Equilibrium Model (MEM)^13^ was reproduced in SLAMM. In the absence of adequate parameterisation of MEM for a given site, the accretion module can simulate a relationship between accretion and elevation. Though, conceptually, the flexible nature of accounting for spatial variability of accretion and ecogeomorphic feedbacks within a wetland allows for greater accuracy in modelling, concern remains around the correct parameterisation of the accretion module for a given site. The accretion module defines accretion in each cell as a function of elevation, salinity and distance to the mouth of the nearest river. The distance to river parameter of the module assumes a linear relationship between sedimentation and the distance of a point from the sediment source, the river channel. Though the basic concept behind such an assumption is not unfounded, it does not hold true for all coastal wetlands where marine influences are greater than riverine. Indeed, sediment input in an estuary, and thereby accretion rates, can vary according to whether riverine, wave or tidal energy dominates the system^5,37^. Thus, use of the distance to channel parameter in the accretion module is questionable, especially when modelling accretion rates for mature barrier estuaries, such as Minnamurra River estuary, where hydrodynamic energy is derived primarily from riverine and tidal processes. Similarly, the relevance of salinity and turbidity maximum zones in mixed estuaries (i.e. Minnamurra River), as opposed to salt-wedge estuaries, on accretion is minimal and was not included in our application of SLAMM.

Care was taken to assign NWI vegetation classes so that they corresponded with mapped vegetation classes at our study site and permitted wetland conversions within SLAMM that correspond to those of the region. SLAMM is conceptualised so that a cell must pass through a predefined sequence of vegetation classes over time in response to SLR. The sequence of vegetation change defined within SLAMM, follows arguments of vegetation succession^48,49^ and subsequent retrogression of zones with transgressing sea levels, as proposed by Bird^50^. Though not strictly in alignment with their original description^39^, NWI categories assigned to wetland vegetation used within the retrospective analyses of this study allowed for vegetation conversions consistent with those observed within barrier estuaries of southeastern Australia. However, with the introduction of a mixed mangrove and saltmarsh category, SLAMM was unable to simulate the retreat of saltmarsh beneath *Casuarina* and dieback of the *Casuarina* forest margins as sea level rose and mangrove encroached, as has been observed in the region^36,51^. In addition, conceptualisation of vegetation change within SLAMM does not adequately allow vegetation to respond to geomorphic change as advocated by many wetland studies in the region^52,53^, particularly where sediment supply to shorelines or shoreline segments is high and able to counteract the effects of SLR. More specifically, SLAMM precludes conversion of water to any vegetation class, despite considerable evidence over the Holocene^53,54^ and more recently^55^, that mangrove shorelines may prograde under conditions of SLR when sediment supply is high. Certainly, given these limitations and potential problems, application of the model to an Australian wetland system and interpretation of its output must be conducted cautiously.

Overall sensitivity of SLAMM to SLR, great diurnal range and salt elevation boundary parameters indicated that the definition and magnitude of inundation were the most important factors affecting SLAMM outputs. Similarly, variations in the accretion parameters, characterised either by rates of SEC, accretion rates or the accretion module, resulted in variations of areal extent for vegetation classes, particularly mangrove and mudflat classes. Errors in the characterisation of these parameters would, therefore, cause possibly significant inaccuracies in model output.

Comparison with other models was only undertaken against deterministic models that were specifically selected to differ from SLAMM on the basis of the underlying numerical model and geographic location for which the model was developed. This criteria meant that model selection was limited and could not be further restricted to those that had undergone rigorous validation. Model output for SLAMM and CM2, a model specifically developed for the Minnamurra site^41^, were relatively similar by the year 2100. As CM2 was developed for the study site, it may be deductive to presume CM2 is the most reliable of the models used in the comparison. CM2 was derived from empirical data collected over relatively short periods of time, and it would have been difficult for the model developers to distinguish significant differences between linear, exponential or polynomial fits to the empirical data. Therefore, it is unreasonable without rigorous validation to presume that CM2 is more reliable than other models. Evidently, the similarities in the outputs from SLAMM and CM2 at 2100 fundamentally relate to the underlying linear and polynomial numerical models used to project substrate elevations over time, and may not be indicative of model reliability.

Treatment of vegetation change in SLAMM precludes shoreline progradation, but this does occur in CM1 and to a lesser extent in CM2. The greater capacity to simulate shoreline progradation in CM1 explicitly relates to the exponential relationship between inundation and SEC upon which the model was founded^40^. While both CM1 and CM2 are derived from the same empirical data and based upon equally robust relationships (CM1: exponential relationship r^2^ = 0.86; CM2: linear accretion relationship r^2^ = 0.73, linear elevation relationship r^2^ = 0.68), the timescale over which data collection occurred (i.e. 10 years) precluded adequate differentiation of the numerical relationship underlying SEC at this study site, and emphasises the need for long-term monitoring of SEC and accretion to improve SLAMM conceptualisation.

All models used in the model comparison were conceptualised upon the assumption that specific vegetation types thrive within certain elevation ranges. Studies in southeastern Australia indicate this is justified^56,57^, though other factors such as substrate physicochemical characteristics may be equally relevant to wetland distribution^36^. The effect of the underlying rules regarding vegetation succession in SLAMM was evident through model comparison.

The initial definition of vegetation distribution and modelling of vegetation change within each model influenced the model output. Significant differences calculated for the initial year of modelling, 2011, were the result of initial vegetation distributions being differentially simulated. CM1 and CM2 define vegetation by discrete elevation ranges from the initial year, whereas SLAMM utilised a vegetation map to ascertain the initial distribution of vegetation. For time zero, vegetation distributions within SLAMM could therefore be considered the most accurate. However, this assumption does not hold true following extrapolation as errors and uncertainty will have been propagated in all models.

The importance of region and site-specific factors and their influence on inundation, SEC and vegetation distribution, as has already been discussed with regards to coastal geomorphology and tidal attenuation, requires additional consideration following model comparison. While SLAMM and CM2 were relatively consistent at 2100, CM2 incorporates other site specific factors such as rainfall and southern oscillation index, which was established following factorial analysis of empirical data. The influence of El Niño Southern Oscillation on rainfall, sea levels and wetland substrate elevations has been well-established in the region^58–60^, and it is reasonable that climatic perturbations be considered within future projections of wetland vegetation in southeastern Australia, and elsewhere. While robust projections of rainfall and SOI to 2100 are not available for the region, efforts to improve global climate models, regional climate models and downscaled climate models will likely enhance the capacity to incorporate these factors within model simulations. As a corollary, SLAMM, CM1 and CM2 did not incorporate stochastic or pulsing events, such as storms and floods that variably influence both surface and subsurface processes within wetlands^61^. While a nearby study found little sustaining effect of a series of east coast low storms and associated flooding on wetland SEC^60^, the stochastic nature of storms and flooding means that further empirical data is required and preclusion of their influence is premature. Stochastic storm events have been reasonably incorporated in probabilistic projections of open coast shorelines in the region^62^. Incorporation of stochastic events within SLAMM, or the comparison models CM1 and CM2, is dependent upon how models are operated and model limitations. However, stochastic events could be considered in future modelling efforts, as has occurred for open coast shorelines.

For the most part, SLAMM and comparison models were characterised by empirical data collected over a relatively short time period. Based on the principle of uniformitarianism, simulations are then conducted over larger time scales. However, the factors and interrelated processes driving wetland evolution over time and, by extension, under rising sea levels occur over a broad range of temporal and spatial scales^63^. For instance, eustatic SLR adjustments influencing the geomorphic changes of coastal wetlands operate at geological timescales whilst pulsing events, such as storms and floods, occur at event scale temporal periods^64^. Both processes are known to affect the evolution of coastal wetlands over time. It is therefore unlikely that the short-duration data used to characterise, and indeed initially parameterise and calibrate SLAMM, and comparison models, is thus able to capture the full suite of interrelated processes influencing the response of wetlands to SLR. Indeed, empirical data associated with rates of SLR as high as those projected to occur in the latter half of this century is not available. Model validation under projected high rates of SLR can therefore not be validated against empirical data and relies entirely upon theoretical validation. In addition, small differences in the initial values of parameters can lead to considerable variation in the model output. In this way, even small errors contained within the measurements used to define SLAMM can produce large errors in output. Considerable care is required, therefore, in obtaining the most reliable and accurate site-specific data prior to the application of SLAMM or indeed any model simulating future wetland vegetation change. In comparing the three models, it becomes clear that the final realisation of vegetation distributions under rising sea levels is affected by error and uncertainty resulting from model conceptualisation and the nature of empirical data. Given the uncertainty of the future and general unpredictability of the dynamic coastal wetland system^65^ it is difficult to ascertain which model could be realistic enough to meet their intended purpose, which in this study was to accurately describe wetland response for wetlands associated with a barrier estuary, such as those of southeastern Australia.

## Methods

### Study site

The Minnamurra River (34°38′S, 150°52′E) is situated on the southeast coast of Australia, approximately 110 km south of Sydney, and drains a relatively small catchment area of 117 km^2^. The coastline of this region is wave-dominated and the Minnamurra River estuary has been classified as a mature, wave-dominated barrier estuary^37^ on the basis of the large sand barrier at its entrance, the degree of estuary infill within the bedrock valley, the significant development of alluvial floodplains within the catchment, and the channelised nature of the river. The tidal and salinity regimes are influenced by the morphology of the estuary entrance, and the channel geometry, resulting in attenuation of tidal flow^38^ and mixed salinity regimes.

Intertidal areas of the Minnamurra River support both mangrove and saltmarsh communities, with broad ecotones where the intertidal slope is low. Extensive stands of mangrove forests, namely *Avicennia marina* in more saline areas and *Aegiceras corniculatum* in the freshwater influenced areas, are observed at lower elevations of the vegetated intertidal zone, whilst saltmarsh is positioned at higher elevations extending to highest astronomical tide level. Saltmarsh species diversity is relatively high and supports a mosaic of species dominated by *Sporobulus virginicus*, *Sarcocornia quinqueflora*, *Samolus repens*, *Suaeda australis* and *Juncus krausii*. Terrestrial vegetation bordering the coastal wetlands is dominated by *Casuarina* forests. In the upper reaches of the river, landward mangrove boundaries merge with *Casuarina* forests, saltmarsh communities being largely absent.

Intertidal vegetation maps have been prepared from aerial photography extending back to 1949^41,51^ and have been augmented by more recent mapping using more advanced imagery^41^ and classification techniques^41,66^. A pattern of mangrove expansion and associated saltmarsh decline has been occurring over the period of aerial photography, and is consistent with a regional trend of mangrove expansion into saltmarsh in southeastern Australia^67^ and mangrove proliferation around Australia^55,68^.

### Conceptual approach

This study aims to validate SLAMM for application at Minnamurra River and compare this model to similar models intended to project the effect of sea-level rise on coastal wetlands. A full description of SLAMM is provided in Supplementary Material 1. Model validation has been widely debated for decades^23,69^. A contributing factor to the problem is the lack of a clear definition of validation as it pertains to the many branches of the modelling community. Acknowledging the difficulty and linguistic complexity in using the term validation, model validation is defined for the purposes of this paper as the process of determining “that a model within its domain of applicability possesses a satisfactory range of accuracy consistent with the intended application of the model”^24^. The domain of applicability encompasses coastal wetlands, specifically for the southeast Australian context, and the processes affecting their evolution with sea-level rise, whilst the intended application of the model is, as it was originally, to reliably study and project the response of these environments to long-term sea level rise. This paper focuses more upon the quantitative aspect of model validation, where predictive ability of a model is determined based upon the agreement of the output data with field data^70,71^. The specific steps undertaken in this study include model set-up, retrospective validation, model comparison and sensitivity analysis.

(i) *Model set-up* is required prior to model validation to ensure that input data sets are calibrated to correspond to conditions at the study site. Model set-up in this study focused on four input parameters, DEM, vegetation classes, tidal attenuation, and SEC, which were calibrated to correspond to empirical data from the study site. As SEC and accretion are not synonymous^45^, and rates of SEC derived from surface elevation tables was available^41^, data calibration of SEC and accretion was not essential and SEC was presumed to be the most valid data set. The full methods and results for model set-up are provided in Supplementary Material 2. Methods for tidal attenuation analysis are provided below as they are pertinent for the study site and subsequent model comparison.
(ii) *Retrospective validation* of SLAMM, as undertaken previously, has been performed over relatively short timescales (up to ~30 years). This study includes two periods of retrospection approximately corresponding to a decade (1986–1997) and half a century (1949–1997), with the intent being to assess its performance over both short and long periods of projection.
(iii) *Model comparison* was undertaken by projecting SLAMM and other models applied to the study site forward. Whilst model comparison does not facilitate an accuracy assessment, and therefore is not a means of model validation, it does provide useful information regarding model structure and its influence on model outputs. Model comparison was undertaken by comparing SLAMM outputs with outputs from a temporally adjusted version of the spatial accretion model developed by Temmerman *et al*.^40^, henceforth termed comparison model 1 (or CM1), and the empirically-derived wetland elevation model developed by Oliver *et al*.^41^ for the study site, henceforth termed comparison model 2 (or CM2). Further details of the models are provided in Supplementary Material 3.
(iv) *Sensitivity analysis*, a built-in component of SLAMM, was undertaken to identify the input variable that had the greatest influence on model outputs.

### Tidal attenuation analysis

As part of model set-up for this study, a method was developed to account for tidal attenuation, and a comparison conducted to examine the importance of considering the process in modelling. Within a region, and within an estuary, site characteristics, such as tidal range and accretion, can vary spatially as tides propagate along channels and across wetlands surfaces that have varying roughness due to the variation in underlying substrates and *in situ* vegetation. The effect of this spatial variation is that tidal amplitude (and accretion) will not be spatially homogenous within an estuary. Modelling of tidal attenuation has been undertaken in the region^38^, and the effect of tidal attenuation on inundation corresponds to landward wetland vegetation boundaries. Such variation can be partially accounted for in SLAMM by dividing the study area into subsites and applying site specific parameters of tidal range to subsites. As accretion can be modelled as proportional to elevation and, by extension, tidal range within SLAMM, identification of accretion parameters specific to each subsite was not required. To account for variance in tidal ranges along the Minnamurra River, eight subsites were identified based upon derived attenuated tidal range values^38^. Subsites approximately correspond to those defined in a prior study^51^. To demonstrate the significance of accounting for tidal attenuation or propagation along an estuary, model outputs produced when using subsites as a proxy for tidal attenuation and SLAMM outputs developed from using a single tidal range value for the entire study site were compared. The relatively basic tidal attenuation model was derived from two data sources, tide gauge data and a digital elevation model, and each has its own uncertainty. The effect of uncertainty in tidal regimes, expressed in tide gauge data, and its representation on landforms, expressed in the DEM, would be compounded in the tidal attenuation model. The authors acknowledge that further validation of this model is required, however patterns of tidal attenuation throughout the model generally corresponded to expected changes in the elevation of vegetation boundaries.

### Retrospective validation

Retrospective validation was undertaken over a short time period corresponding to a decade (1986–1997) and half a century (1949–1997), longer than previous retrospective validations of SLAMM. Preparation and refinement of input parameters are detailed in Supplementary Material 1. Historical vegetation maps^51^ were used as the input vegetation information for validation runs and as real-world vegetation distribution for model comparison. Accretion can be modelled in a variety of manners within SLAMM version 6.2. To best emulate the processes and feedbacks mechanisms occurring within a wetland system, the accretion module, which accounts for spatial and temporal variability of accretion, was implemented within this study. Furthermore, rates of SEC were used in place of accretion rates to define the accretion module in order to account for both positive and negative gains in wetland elevation over time. Though net wetland SEC over time is controlled by a number of dynamic processes, including accretion and compaction^46^, only accretion is modelled within SLAMM. Thus, use of rates of SEC, which implicitly account for autocompaction and other processes contributing to decreases in wetland surface elevation, is considered necessary to more accurately reflect wetland evolution over time and potentially prevent overestimation of the ability of coastal wetlands to build vertically under rising sea levels.

Given the lack of accurate elevation information for the relevant input years, DEM2 was adjusted within SLAMM using the NAVD88 correction parameter to produce an elevation surface representative of the required initial year of validation runs. The NAVD88 correction parameter is included in SLAMM to adjust elevation data from a height datum to a tidal datum^9^. With rising sea levels there is logically a corresponding rise in the local tidal datum. Thus, based on the observed SLR at Port Kembla of 2.1 mm/yr and Minnamurra mean sea level^72^, elevation adjustments were defined by extrapolating the degree of SLR occurring over the period of retrospection and subtracting this value from Minnamurra mean sea level.

Following retrospection of the DEM2 to 1949 and 1986, the new DEMs were then used as SLAMM input data. Projections were undertaken using the best available input parameters, as identified through the model set-up phase. SLAMM projections for the period 1986–1997 were visually and statistically compared to the 1997 mapped vegetation distribution^51^. Similarly, for the period 1949–1997, SLAMM projections were compared to the 1963, 1986 and 1997 mapped vegetation distributions. Statistical analysis was performed to evaluate the fit of the modelled data to the available historical data and to test if errors associated with model outputs were within acceptable limits. Deviations in the area of vegetation classes of less than 10% from observed vegetation distribution for the initial year of modelling were considered statistically valid. Errors were also examined for the final year of modelling (i.e. 1997). Since statistical tests were based purely on numerical deviations in area, further visual and spatial analysis, using the raster calculator tool in the ArcGIS spatial analysis extension, accompanied any statistical analysis to ensure that modelled spatial distributions of vegetation were indeed similar to those observed.

### Model comparison

Models were selected for comparison with SLAMM on the basis of a number of criteria pertaining to the location for which the model was developed and parameterisation, and the underlying mathematical function upon which the model behaves (Table 2). These models and their parameterisation for model comparison are described below.

**Table 2:** Conceptual differences between SLAMM and other models. Conceptual differences were used as the criteria for selection of comparison models.

Model Conceptualisation	SLAMM	CM1	CM2
Location developed	U.S.A.	Scheldt Estuary, Netherlands/Belgium	Minnamurra River, southeastern Australia
Primary parameters	- Elevation - Slope - Tidal Range	- Elevation - Distance to Channel	- Elevation - Distance to Channel - Rainfall - SOI
SEC Model: Accretion + Subsidence	Polynomial	Exponential	Linear
Vegetation Model	Based on the *lower* boundary	Based on the *upper* and *lower* boundary	Based on the *upper* and *lower* boundary

#### SLAMM

Model description and parameterisation is provided in Supplementary Material 1. SLAMM was developed for marshes associated with the North Atlantic Ocean. Few attempts have been made to validate and calibrate SLAMM in regions beyond the North Atlantic Ocean. SLAMM is now readily available and widely applied. The underlying mathematical function driving wetland surface elevation changes (SEC) is based upon a polynomial relationship between a range of variables.

#### CM1

A relationship between sedimentations rates and a number of controlling morphometric parameters on saltmarsh substrates along the Scheldt estuary has been developed^40^. The point-based, empirical relationship was applied spatially to simulate the varying patterns of sedimentation over the entire platform. The underlying mathematical function driving SEC is based upon an exponential relationship between a range of variables. A method was developed to convert this empirical model to a temporally dependent spatial model that could be applied using empirical data from our study site within ArcGIS. See Supplementary Material 3 for full description of model development and parameterisation.

#### CM2

An empirically based spatial model that simulates wetland surface elevation and vegetation distribution under conditions of rising sea levels was developed specifically for the study site using empirical data from the study site^41^. Whilst CM2 could initially be expected to be the most reliable model, the model developers recognise that the model outcomes are highly dependent on assumptions and input parameters. In addition, retrospective validation of CM2 has not been undertaken. For this reason, no assumptions regarding its expected reliability are made during the model comparison. The model was based on the significance of catchment based variables at Minnamurra River in an initial stepwise regression analysis of accretion and surface elevation trends derived from empirical surface elevation table-marker horizon (SET-MH) data of accretion and SEC^41^. Subsequent factorial analysis of variance established the best fit between catchment variables and site-specific accretion and surface elevation trend data. The underlying mathematical function driving SEC was based upon a linear relationship between factors considered significant in the model and SEC, including time (i.e. days from first SET measurement), average rainfall for the previous month, 6-month average water level, distance to the shore, 3-month averaged southern oscillation index value and the mean sea level. This function was applied in ArcGIS at decadal time increments.

#### Comparison technique

**Figure 4:**
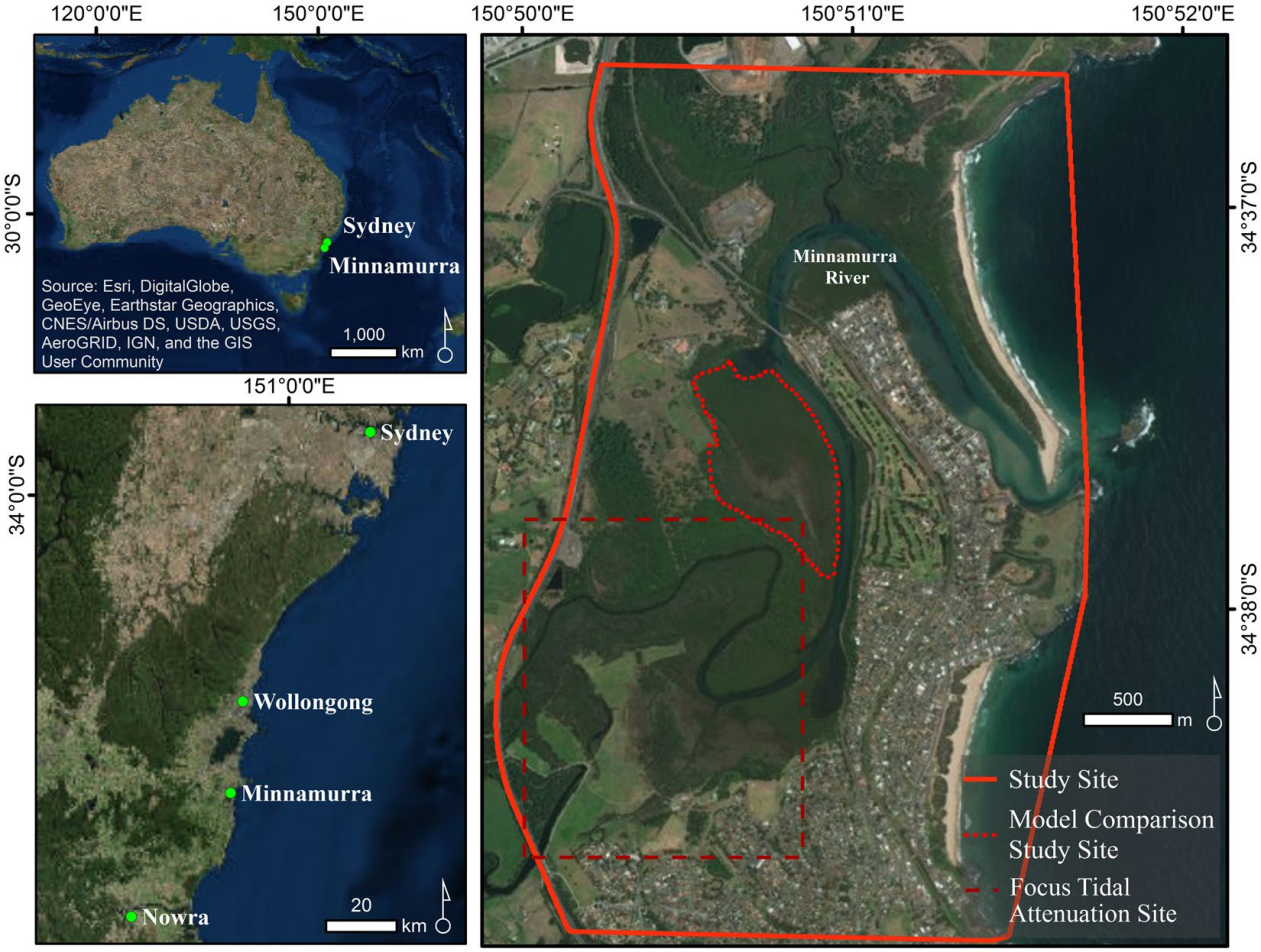
Location of the study site, Minnamurra River, where SLAMM data validation, sensitivity analysis and retrospective analyses were undertaken. Model comparison was undertaken within a subset of this broader study site. (Figure generated using ArcMap Version 10.4.1).

The model comparison study site lies on the western floodplain of the Minnamurra River, approximately 3 km upstream from the river entrance (Fig. 4). This site was selected as it corresponded to the location for which CM2 was specifically developed and was a region for which model parameters could be relatively consistently applied to all models, ensuring reasonable comparability was possible. Parameters consistent across models included the consideration of SLR scenarios. Initially a high and low SLR scenario was selected based on the B1 and A1FI SLR scenarios, as described in the IPCC fourth assessment report^73^. As differences between model outputs were likely to be more evident under a high SLR scenario, this study presents outputs derived from the A1FI (95%CI) SLR scenario only. Based upon data calibration and consistency in application between models, DEM3 elevation information were utilised in the comparison process. Surface elevation and accretion trends were derived from SET-MH data at Minnamurra River and rates of accretion and SEC were consistently applied in all models. Vegetation elevation parameterisation was based upon previously described elevation ranges^41^ and was consistently applied in all models. The initial vegetation starting conditions differ between models. SLAMM application describes vegetation classes as a function of a cell’s elevation, whilst comparison models assign vegetation on the basis of initial vegetation extents derived from maps. Whilst these initial vegetation conditions differ between models, the approach for comparison is consistent with the validation approach as it ensures that model application occurs as intended. Consequently each model has been applied according to that intended in technical documentation. Despite differing initial vegetation conditions, comparison at 2100 is reasonable as each model has been applied as intended.

Visual and statistical comparison of the simulated output of the different models was conducted. Differences and similarities between model outputs identified were examined closely to determine the source of variations. In doing so, the scientific principles and assumptions upon which each model was based were considered. In addition, the treatment of influential factors, the mathematical definition and conceptual abstraction of the wetland system were examined and compared in an attempt to evaluate the performance of the models and determine if a specific model was more appropriate for the simulation of Minnamurra wetland evolution with rising sea levels.

### Sensitivity analysis

Sensitivity analysis facilitates the evaluation of the structure of a model. It is achieved by systematically varying model parameters to examine the effect the variance has on model outputs and provides information on the approximate contribution of parameters to model uncertainty^74^. Sensitivity analysis is a built-in function in SLAMM and for this study was performed by iteratively varying selected parameters by ±10% while keeping all other parameters unaltered^9^. Parameters chosen for analysis included great diurnal tidal range, historic trend in SLR, NAVD88-MSL value, salt elevation, rates of SEC in mangrove and saltmarsh, and amount of SLR by 2100. A sensitivity statistic for individual parameters analysed was calculated by relating the percentage change in the parameter (10%) to the proportional change in individual vegetation types modelled for the year 2100. Results were used to investigate the robustness of simulations, to improve understanding of the behaviour of the model and the contribution of parameter error or variation to model uncertainty.

## Electronic supplementary material

Supplementary Information
