# Supplementary material for: Validation and Comparison of a Model of the Effect of Sea-Level Rise on Coastal Wetlands
Source: Sci Rep. 2018 Jan 22;8:1369. doi: 10.1038/s41598-018-19695-2 (PMC5778053; doi:10.1038/s41598-018-19695-2)
Supplement: Supplementary file 1 — Supplementary Information [file 41598_2018_19695_MOESM1_ESM.doc]

VALIDATION AND COMPARISON OF A MODEL OF THE EFFECT OF SEA-LEVEL RISE ON COASTAL WETLANDS

SUPPLEMENTARY MATERIAL 1-3

Mogensen LA^1^

Rogers K^1^, *

1 School of Earth and Environmental Science, University of Wollongong, Wollongong, NSW, 2522 Australia

* Corresponding author: [Kerrylee@uow.edu.au](mailto:Kerrylee@uow.edu.au)

SUPPLEMENTARY MATERIAL 1: SLAMM description

The Sea Level Affecting Marshes Model (SLAMM) is a complex, non-hydrodynamic model that attempts to simulate the response of wetlands to sea-level rise (SLR)^1^. Abstraction of the wetland system response resulted in the development of six processes being included in SLAMM v.6.2, the version used in this study. Inundation, accretion, overwash, soil saturation, erosion and salinity are all included as the primary processes that affect wetland fate under scenarios of SLR. Certain processes are optionally incorporated in simulations, such as overwash and soil saturation, and still others remain in their formative stages and have been recommended not to be utilised, such as the salinity module.

The conceptual model upon which SLAMM is designed is based on the assumption that wetland categories only inhabit a certain elevation range that are a function of tidal range or salinity. The model can simulate changes in 25 different land-cover categories under rising sea levels. Wetland categories are based on the National Wetland Inventory prepared for the United States of America^2^. The structure of the model can be broken into two broad areas pertaining to the wetland elevation change with rising sea levels and the subsequent conversion of elevation-defined vegetation. SLAMM divides an area into cells of a custom-defined size and carries out calculations and conversions on a cell-by-cell basis. The change in wetland surface elevation of a single cell is a function of SLR and accretion, and is defined mathematically as:

|  | 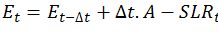 | (1) |
| --- | --- | --- |

Where: *E* is elevation, *A* is the site-specific accretion or sedimentation rate, *SLR* is the SLR for a given time step, and *t* is time in years. The accretion rate can be characterised by vegetation-specific values or may be defined as a function of elevation, distance to channel and/or salinity using the accretion module. The magnitude of SLR follows the IPCC scenarios of the third assessment report (TAR)^3^ or a custom-defined SLR. In addition to the global SLR, the local SLR is simulated from the deviation of the local historic SLR trend from the eustatic SLR trend, assuming a linear relationship is consistent over time. The SLR is therefore calculated at each time step as:

|  | 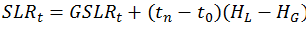 | (2) |
| --- | --- | --- |

where *GSLR* is the global mean *SLR* (m) over a certain time step as custom-defined or following the TAR scenarios, *H_L_* is the local historic trend of SLR (m) and *H_G_* is the eustatic trend of SLR (m). The combination of estimated SLR and accretion responses thus drives the elevation change of a wetland with respect to mean sea level.

Subsequent conversion of a wetland category in a cell is driven by the cell’s elevation. Each category is assigned a specific elevation, salinity or tidal range within which the particular wetland can exist. In any given simulation, if the elevation of the cell falls below the elevation range defined for the wetland category contained in the cell, then a fraction of the cell is converted to a lower-elevation habitat. The fraction of the cell lost is a function of the slope of the land and the magnitude of the fall below the wetland-category’s lowest elevation. The lower the cell falls, the greater the fraction converted to a lower wetland category. Conversions, thus, occur in one direction only, from one wetland vegetation type to another of a lower elevation range. The lower vegetation type to which a category is converted is governed by a decision-tree process programmed into SLAMM from which site-specific deviations cannot occur unless the coding is altered to do so.

SLAMM requires a variety of spatial and site-specific parameters to be determined prior to its implementation. Supplementary Figure 1 provides a general overview of the processes followed and information gathered for the most accurate implementation of SLAMM at the study site. Supplementary Table 1 outlines the input variables and data sources used in this study to parameterise the model.


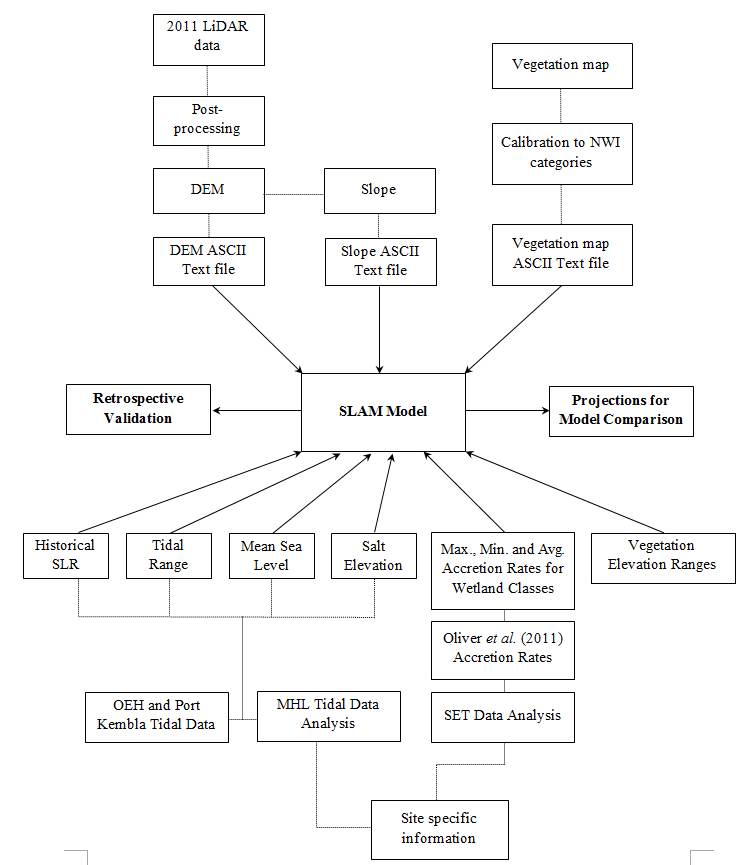


Supplementary Figure **1**: Conceptual diagram of the input parameters to SLAMM for this study.

Supplementary Table 1: Description of input data sets used to parameterise SLAMM in this study.

| Data type | Input  Data | Description |
| --- | --- | --- |
| ASCII text file | DEM^4^ | High-vertical resolution digital elevation model derived from Lidar survey collected as part of the Coastal Capture program spanning the coastal area between Nowra and Wollongong. The Lidar data were collected with an ALS50-II sensor (Leica Geosystems, Heerbrugg, Switzerland) at a flying height of 2000 m, which yielded a nominal nadir point density of 1.03m and an overall vertical and horizontal accuracy of 80cm and 30cm respectively. Data were projected in metres (Geocentric Datum of Australia 1994, Map Grid of Australia Zone 56) with elevation values referenced to the Australian Height Datum 1971 (AHD71). The Lidar data was distributed in the popular format of the *.las file. At the time of obtainment in 2015, the data had been pre-processed, meaning that ground points had been classified from the raw Lidar data. The generated DEM has a horizontal resolution of 5 m and was interpolated using the ‘Natural Neighbours’ interpolation in ArcMap. |
|  | Slope | Derived from the DEM, producing a layer of corresponding spatial extent and spatial resolution. |
|  | Vegetation^5,6^ | Vegetation maps compiled for the years 1949, 1963, 1986 and 1997^5^ were used in SLAMM for various stages of calibration and validation. Vegetation distributions at 2011 for model comparison were derived from subsequent object-oriented vegetation mapping^6^.Vegetation classes were calibrated to correspond to NWI classes. |
| Site specific values | Historical SLR^7,8^ | Used to determine the differential rise in local sea level with respect to the established eustatic SLR of 1.7 mm/y (IPCC 2007). Derived from the Port Kembla and Fort Denison tide gauges, where the temporal period of tidal data collection extended beyond the 18.6 year tidal epoch. |
|  | Tidal range^7^ | The singular value of great diurnal tidal range sourced was used. During model set-up, tidal subsites, primarily identified for their variations in tidal range, were thus each assigned a tidal range based upon the extrapolation of tidal risk data using the Oregon State University Tidal Inversion Software^9^. |
|  | Mean sea level^7^ | An annual average value drawn from MHL^7^ was used to adjust elevations such that local mean sea level was set to zero. |
|  | Salt elevation^7^ | This boundary between inundated and dry lands was defined as the high high water solstice springs (HHWSS). Logically, as the tide range attenuates, so too does the salt elevation boundary. The standard deviation of HHWSS was used to determine an estimate of the upper and lower limit of the salt elevation boundary for model set-up purposes. Given the lack of long term data recorded in the upper reaches of the estuary, the highest value was assigned to the first subsite, closest to the ocean, and the lowest to the final subsite with areas in between being set a value proportional to their distance from the ocean. |
|  | Accretion rates^10^ | Empirical data of surface elevation change (SEC) and accretion were derived from a network of surface elevation tables (SET) and marker horizons (MH) established in mangrove and saltmarsh at the study site, as previously described^10^. A relationship between elevation and accretion rates was derived by calibrating the SLAMM accretion curve to the model of Temmerman, et al. ^11^ using the SEC empirical data. To justify this process an analysis of the relationship between SEC and absolute wetland elevation was undertaken . This analysis indicated that both linear and exponential fits were variably suitable; however the exponential fit was more consistently robust, as indicated in Supplementary Table 2. This model defined the spatial variability of SEC across the wetland and enabled identification of key input parameters of maximum and minimum accretion rates for each wetland vegetation type and cubic equation coefficients that define the curve relating varying accretion rates to elevation. The model set-up procedure also explored constant rates of SEC and constant rate of accretion variables for mangrove, mixed and saltmarsh communities. The outcomes of this process are not reported within this study. |
|  | Vegetation elevation ranges^10^ | Vegetation classes within SLAMM were each assigned elevation ranges within which they exist on the basis of previously defined ranges^10^ and converted to the required half tide units (HTU). |

Supplementary Table 2: Analysis of the exponential and linear fit of SEC data at each time step that data was available.

| Year | Regression type | Equation | R-squared |
| --- | --- | --- | --- |
| 2002 | Exponential | y = 589.69e^-0.147x^ | 0.5351 |
|  | Linear | y = -75.615x + 606.64 | 0.4877 |
| 2003 | Exponential | y = 529.13e^0.0566x^ | 0.0741 |
|  | Linear | y = 34.559x + 552.83 | 0.0956 |
| 2009 | Exponential | y = 541.93e^0.0298x^ | 0.3468 |
|  | Linear | y = 14.652x + 563.5 | 0.295 |
| 2010 | Exponential | y = 644.16e^-0.052x^ | 0.6546 |
|  | Linear | y = -27.657x + 655.07 | 0.6291 |
| 2011 | Exponential | y = 745.19e^-0.057x^ | 0.81 |
|  | Linear | y = -31.412x + 737.97 | 0.8235 |
| 2013 | Exponential | y = 602.37e^-0.024x^ | 0.0724 |
|  | Linear | y = -11.256x + 611.23 | 0.0529 |

References

1 Clough, J., Park, R., Propato, M., Polaczyk, A. & Fuller, R. SLAMM 6.2 Technical Documentation. (2012).

2 Cowardin, L. M., Carter, V., Golet, F. C. & LaRoe, E. T. Classification of wetlands and deepwater habitats of the United States. (U.S. Department of the Interior Fish and Wildlife Service, Washington, D. C., 1979).

3 IPCC, T. W. Climate Change 2001: The Scientific Basis, Contribution of Working Group I to the Third Assessment Report of the Intergovernmental Panel on Climate Change. (Cambridge University Press, 2001).

4 Land and Property Information. (ed New South Wales Government) (Sydney, 2011).

5 Chafer, C. J. A spatio-temporal analysis of estuarine vegetation change in the Minnamurra River 1938-1997. 47pp, plus appendices (Wollongong, 1998).

6 Owers, C. J., Rogers, K. & Woodroffe, C. D. Identifying spatial variability and complexity in wetland vegetation using an object-based approach. *International Journal of Remote Sensing* **37**, 4296-4316 (2016).

7 MHL. OEH NSW Tidal Plane Analysis: 1990-2010 harmonic analysis. (2012).

8 Couriel, E., Modra, B. & Jacobs, R. in *17th Australian Hydrographers Association Conference* (Sydney, 2014).

9 Oregon State University Regional Tidal Solutions (http://volkov.oce.orst.edu/tides/PO.html, 2013).

10 Oliver, T., Rogers, K., Chafer, C. & Woodroffe, C. Measuring, mapping and modelling: an integrated approach to the management of mangrove and saltmarsh in the Minnamurra River estuary, southeast Australia. *Wetlands Ecology and Management* **20**, 353-371, doi:10.1007/s11273-012-9258-2 (2012).

11 Temmerman, S., Govers, G., Wartel, S. & Meire, P. Spatial and temporal factors controlling short-term sedimentation in a salt and freshwater tidal marsh, Scheldt estuary, Belgium, SW Netherlands. *Earth Surface Processes and Landforms* **28**, 739-755, doi:10.1002/esp.495 (2003).

SUPPLEMENTARY MATERIAL 2: Model set-up

Model set-up was undertaken to ensure that input data sets were calibrated to correspond to conditions at the study site. This study focused on three input parameters, digital elevation model (DEM), vegetation classes and tidal attenuation, which were calibrated to correspond to empirical data from the study site. This supplementary material provides the methods and results for DEM and vegetation calibration. Methods and results for tidal attenuation calibration are provided in the main text.

Model set-up methods

*DEM calibration*

Since the topography of the land determines the potential frequency and magnitude of inundation, high-resolution elevation data is arguably the most important component when modelling the effect of sea-level rise (SLR) on coastal wetlands ^1,2^, and calibration of DEMs against empirical data of wetland elevations is an essential model set-up step to be undertaken prior to model projections. Pre-processed Lidar data (as a *.las file) and additional elevation data were used to generate three DEMs, including:

1. DEM1, generated using pre-processed surface height information, applying a 5 m resolution, natural neighbour interpolation, and correcting the influence of the bridge over the Minnamurra River on the DEM;
2. DEM2, generated by correcting DEM1 on the basis of filtering incorrectly classified ground point data, calibrating data against 210 Real Time Kinematic-Global Positioning System (RTK-GPS) derived elevation points (1-5cm vertical accuracy and mean horizontal accuracy of 1 cm), and validating the derived-DEM against an additional 72 RTK-GPS derived-elevation points
3. DEM3, generated using a combination of selected Lidar data points that were deemed reliable and intersected vegetation units, and 300 RTK-GPS derived elevation points, that were interpolated using weighting interpolation techniques (IDW) to a horizontal resolution of 5 m.

DEM accuracy assessment was then undertaken in the model comparison subsite of the study area. The 30% subset (72 points) of the RTK-GPS points collected previously^3^ were used as the ground control points from which elevation errors were measured. DEM-derived elevations were identified for each of the RTK-GPS points, and the following variables were then determined for each DEM: root mean square error (RMSE), maximum, minimum, mean and skewness of errors. As vertical accuracy of Lidar data is often dependent upon the land-cover and vegetation of a particular area^2,4^, the analysis was also undertaken on the basis of vegetation type, which included mangrove, saltmarsh and *Casuarina*.

*Vegetation calibration*The Sea Level Affecting Marshes Model (SLAMM) can simulate changes in 25 different land-cover categories under rising sea levels. Wetland categories are based on the National Wetland Inventory (NWI), originally prepared for applications in the United States of America^5^. These vegetation communities do not directly correspond to the vegetation communities of coastal wetlands of southeastern Australia. Consequently, vegetation category calibration, whereby appropriate NWI categories were assigned to southeastern Australian vegetation communities, was essential before undertaking any modelling using SLAMM. NWI categories were assigned based upon the approximate similarity of an Australian wetland vegetation to a vegetation description by Cowardin, et al. ^5^and, most importantly, the conversion of the vegetation that would be simulated with inundation in SLAMM. Some emphasis was placed on the latter when determining corresponding NWI categories for *Casuarina* and saltmarsh communities.

Recent changes in vegetation distribution documented within the region^6,7^ indicate that boundaries between vegetation classes are dynamic and often poorly defined as they are not characterised by abrupt or linear changes in vegetation. Rather, boundaries between communities are gradual with mixed community ecotones developing, particularly between mangrove and saltmarsh, but also occurring between saltmarsh and *Casuarina*. However, as mixed zones were not a class included in prior mapping^8^, ecotones were not identified nor incorporated in vegetation calibration for the retrospective validation since inclusion would only serve to increase error between modelled and observed (mapped) vegetation distributions. Consequently, the seven classes within prior mapping data^8^ were reclassified to correspond to the most suitable NWI categories. For model comparison, however, a mixed ecotone of mangrove and saltmarsh was modelled in SLAMM due to the category’s inclusion within both the comparison model 1 (CM1) and 2 (CM2). Given the addition of a category into the modelling process, vegetation classes were again reclassified to correspond to the most suitable NWI categories.

Model set-up results

*DEM calibration*

Results from DEM calibration indicated that further post-processing of Lidar data improves the vertical accuracy of derived elevation surfaces within wetland areas. DEM1, derived from as-received Lidar data, produced a global accuracy of 0.42 m. Vertical accuracy, however, was found to be spatially variable. Whilst saltmarsh zones of DEM1 recorded an increased accuracy in elevation when compared to the global accuracy, significant vertical errors occurred within mangrove areas (Supplementary Table 3) where denser vegetation predominated. Vertical errors in mangrove zones corresponded spatially with misclassified Lidar points, specifically where lower branches of mangrove had been classified as ground within the as-received dataset.

Further post-processing of the Lidar data resulted in a considerable increase in the accuracy of surface elevations within wetland areas (Supplementary Table 4). A notable decrease in elevation errors within mangrove areas of DEM2 was recorded, whilst the elevation accuracy of saltmarsh zones remained identical to that calculated for DEM1. Though elevations of wetland areas included fewer errors, some inaccurate representations of surface elevation remained, particularly along the river bank within the upper reaches of the estuary. The limitations of Lidar systems and classification algorithm applied are considered primarily responsible for the propagated errors evident within DEM2.

Supplementary Table 3: Vertical accuracy statistics for DEM1 derived from the as-recieved Lidar data.

| Land Cover | No. of Points | RMSE_Z_ (m) | Mean (m) | Minimum (m) | Maximum (m) | Skew | Standard Deviation (m) |
| --- | --- | --- | --- | --- | --- | --- | --- |
| All | 72 | 0.42 | 0.27 | -0.62 | 1.21 | 0.96 | 0.32 |
| Mangrove | 22 | 0.68 | 0.54 | -0.62 | 1.21 | -0.63 | 0.42 |
| Mixed | 30 | 0.23 | 0.16 | -0.28 | 0.81 | 1.44 | 0.17 |
| Saltmarsh | 12 | 0.20 | 0.15 | -0.01 | 0.51 | 1.80 | 0.13 |
| Casuarina | 8 | 0.24 | 0.14 | -0.15 | 0.54 | 0.95 | 0.21 |

Supplementary Table 4: Vertical accuracy statistics for DEM2, developed following further post-processing of the as-received Lidar data.

| Land Cover | No. of Points | RMSE_Z_ (m) | Mean (m) | Minimum (m) | Maximum (m) | Skew | Standard Deviation (m) |
| --- | --- | --- | --- | --- | --- | --- | --- |
| All | 72 | 0.31 | 0.15 | -0.62 | 1.65 | 2.51 | 0.28 |
| Mangrove | 22 | 0.45 | 0.20 | -0.62 | 1.65 | 2.06 | 0.42 |
| Mixed | 30 | 0.22 | 0.12 | -0.30 | 0.80 | 1.34 | 0.19 |
| Saltmarsh | 12 | 0.20 | 0.15 | -0.01 | 0.51 | 1.80 | 0.13 |
| Casuarina | 8 | 0.26 | 0.13 | -0.29 | 0.54 | -0.06 | 0.24 |

*Vegetation calibration*

Assignment of NWI categories to vegetation communities of southeastern Australia, specifically occurring at Minnamurra are presented in Supplementary Table 5. These seven categories, corresponding to the classes previously mapped^8^, were utilised for the retrospective validation within this study. Though it is recognised that *Casuarina* does not completely fit the description of the NWI category assigned, the *Tidal swamp* category was chosen to allow for conversion of *Casuarina* to saltmarsh within SLAMM. *Transitional Salt Marsh*, the NWI category to which undeveloped land will convert, was considered in this study to represent the potential expansion of *Casuarina* into undeveloped areas and was included as such in calculations of model error within the retrospective validation of this study.

Supplementary Table 5: Reclassification of previously mapped vegetation classes^8^, as defined in this study, to match NWI vegetation classes coded within SLAMM.

| Vegetation class^8^ | NWI vegetation class | SLAMM ID code | Under inundation, converts to |
| --- | --- | --- | --- |
| Mangrove | Regularly Flooded Marsh | 8 | Mudflat (Tidal Flat) |
| Saltmarsh | Irregularly Flooded Marsh | 20 | Regularly Flooded Marsh |
| Casuarina | Tidal Swamp | 23 | Irregularly Flooded Marsh |
| Undeveloped Land | Undeveloped Dry Land | 2 | Nearest Transitional Salt Marsh, Mangrove, Ocean or Estuarine Beach |
| Minnamurra River | Estuarine Open Water | 16 | N/A |
| Ocean | Open Ocean | 19 | N/A |
| Sandy Beach | Ocean Beach | 12 | Ocean |

Model comparison conducted within this study incorporated the modelling of a mixed ecotone of mangrove and saltmarsh. The NWI categories assigned to each vegetation community and the mixed ecotone are presented in Supplementary Table 6. Beach and ocean categories did not form part of the comparison study site and, as such, are not included within Supplementary Table 6.

*Supplementary Table 6: NWI categories assigned to southeastern Australian vegetation classes and the mixed ecotone of mangrove and saltmarsh to be used within SLAMM for model comparison.*

| Vegetation class | NWI vegetation class | SLAMM ID code | Under inundation, converts to |
| --- | --- | --- | --- |
| Mangrove | Regularly Flooded Marsh | 8 | Mudflat (Tidal Flat) |
| Mixed | Irregularly Flooded Marsh | 20 | Regularly Flooded Marsh |
| Saltmarsh | Tidal Swamp | 23 | Irregularly Flooded Marsh |
| Casuarina | Transitional Salt Marsh | 7 | Regularly Flooded Marsh |
| Undeveloped Land | Undeveloped Land | 2 | Nearest Transitional Salt Marsh, Ocean or Estuarine Beach |
| Minnamurra River | Estuarine Open Water | 16 | N/A |

References

1 Morris, J. T. *et al.* Integrating LIDAR elevation data, multi‐spectral imagery and neural network modelling for marsh characterization. *International Journal of Remote Sensing* **26**, 5221-5234, doi:10.1080/01431160500219018 (2005).

2 Schmid, K. A., Hadley, B. C. & Wijekoon, N. Vertical Accuracy and Use of Topographic LIDAR Data in Coastal Marshes. *Journal of Coastal Research* **275**, 116-132, doi:10.2112/jcoastres-d-10-00188.1 (2011).

3 Oliver, T., Rogers, K., Chafer, C. & Woodroffe, C. Measuring, mapping and modelling: an integrated approach to the management of mangrove and saltmarsh in the Minnamurra River estuary, southeast Australia. *Wetlands Ecology and Management* **20**, 353-371, doi:10.1007/s11273-012-9258-2 (2012).

4 Flood, M. ASPRS Guidelines: Vertical accuracy reporting for LiDAR data. *Amer. Soc. Photogram. Remote Sens. LiDAR Committee. Ver* **1** (2004).

5 Cowardin, L. M., Carter, V., Golet, F. C. & LaRoe, E. T. Classification of wetlands and deepwater habitats of the United States. (U.S. Department of the Interior Fish and Wildlife Service, Washington, D. C., 1979).

6 Saintilan, N. & Williams, R. J. Mangrove transgression into saltmarsh environments in south-east Australia. *Global Ecology and Biogeography* **8**, 117-124 (1999).

7 Saintilan, N., Wilson, N., Rogers, K., Rajkaran, A. & Krauss, K. W. Mangrove expansion and salt marsh decline at mangrove poleward limits. *Global Change Biology* **20**, 147-157 (2014).

8 Chafer, C. J. A spatio-temporal analysis of estuarine vegetation change in the Minnamurra River 1938-1997. 47pp, plus appendices (Wollongong, 1998).

SUPPLEMENTARY MATERIAL 3: Description and development of CM1 and CM2

This supplementary material provides an in depth description of the development of Comparison Model 1 (CM1) and the method by which it was implemented for this study. The model is based upon an adjusted form of an empirically-based, spatial model^1^. Model description of CM2 is also provided within this supplementary material.

Comparison Model 1

*Model description and development*

A relationship between sedimentation rates and a number of controlling morphometric parameters on saltmarsh substrates along the Scheldt estuary has been established^1^. The point-based, empirical relationship was applied spatially to simulate the varying patterns of sedimentation over the entire platform. Based on the common understanding that environmental processes are interconnected and can act synergistically, the sedimentation rate of each cell-defined point on the saltmarsh platform was calculated using the equation:

| 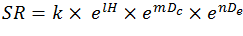 | (1) |
| --- | --- |

where *SR* is the sedimentation rate, *H* is the intensity of tidal inundation estimated as the surface elevation with respect to the mean high tidal water, *D_c_* is the linear distance to the nearest tidal channel, *De* is the distance to the marsh edge measured along the closest tidal creek and *k, l, m,* and *n* are parameters estimated by a multiple non-linear regression procedure. Since sedimentation is a process that contributes to the overall change in wetland surface elevation, it was considered justifiable to replace the original dependent variable of the model^1^, sedimentation rate, with surface elevation change (SEC).

As coastal wetlands of southeastern Australia are comprised of both saltmarsh and mangrove communities, the exponential relationships observed on the saltmarsh substrates along the Scheldt Estuary are not necessarily present along southeastern Australian estuaries, such as Minnamurra River. To counteract this potential problem, empirical relationships between model variables were analysed to ensure the existence of an exponential relationship prior to the application of this adjusted spatial model^1^ to coastal wetlands of the Minnamurra River. Unlike the saltmarsh environment of northwestern Europe (Belgium/The Netherlands), the southeastern Australian coastal wetlands do not characteristically have distinct channel networks that act as conduits for water flow to a tidal channel. Instead, ebb and flow of tides occur in a relatively sheet flow manner. This being the case, the parameter, distance to edge, used in the original model^1^ was set to zero in its application here, effectively excluding the variable from the model.

The adjusted model resulting from the above modifications was used as a parameter in modelling the wetland surface evolution to the end of the century. Evolution of coastal wetlands is inherently time dependent and is the natural result of the system’s response to changes in external conditions^2,3^. Changes in sea level, itself a response to changing external conditions, therefore has a significant influence on the evolution of coastal wetlands. The adjusted model, as a surrogate of the system’s response to change, was thus used in combination with estimated sea-level changes modelled by the Intergovernmental Panel on Climate Change^4^ to simulate wetland evolution to the year 2100. Following the zero-dimensional models from previous research^2,5-8^ and maintaining wetland surface heights relative to mean high water (MHW) as required by the adjusted model, elevations at a given timestep were calculated as:

| 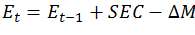 | (2) |
| --- | --- |

where *E_t_* is the elevation at a given time step, *E_t-1_* is the elevation at the previous time step, *SEC* is the surface elevation change as estimated by Equation 1 and *ΔM* is the incremental rise in sea level. This equation formed the elevation component of Comparison Model 1 (CM1) and represents the geomorphic component of the model.

Parameterisation of the model required information on *H* (intensity of tidal inundation), *D_c_* (linear distance to the nearest tidal channel), and model coefficients (*k, l, m*). Statistical analysis for model coefficients was determined using a non-linear regression in JMP, and required time series information of *H* and *D_c_*. Methods for generating time series of *H* and *D_c_* are below:

- *To produce a time series of H*: the local mean high water (MHW) level recorded at the Minnamurra River was subtracted from the final elevation values calculated at individual surface elevation tables (SETs) established at the study site. To capture the tidal conditions of each year as truly as possible, the yearly-averaged MHW levels of the Minnamurra River as reported by MHL^9^ were used for the period 2003-2010, a temporal period corresponding to the establishment of the tidal gauge at the Minnamurra River and final year analysed in the MHL^9^ report. For years not within 2003-2010, an eight-year time-averaged MHW level, as reported by MHL^9^, was used. Given that the small variabilities in MHW data displayed no significant trend over time, the constant, averaged value was considered to be sufficiently representative of the MHW level at the Minnamurra site. To ensure this was the case, further analysis was undertaken.
- *To obtain the Dc parameter:* the distance from each SET to the Minnamurra River was calculated using the ‘Euclidean Distance’ tool in ArcMap. Final elevation and distance to channel information were recorded before being exported to JMP Pro (SAS v.11).

Each regression model was set to iterate 1,000,000 times or until convergence was reached. The fit of the modelled data to the established elevation trends was calculated using a linear-regression. Factors for analyses included absolute elevations relative to MHW and distance to channel values. Coefficient of determination (i.e. r^2^) greater than 0.85 was deemed to provide sufficient explanation of the observed data. The equation and coefficients (*k, l, m*) deemed to provide the most adequate explanation of the wetland system was identified as:

| 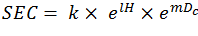 | (3) |
| --- | --- |

where *k* = 0.00112762606907659, *l* = -5.12615410987054, and *m* = -0.00378438213262039.

*Model implementation*

Equation 2 was applied at decadal intervals to grid-based elevation surfaces in ArcMap to simulate wetland elevation change as a response to SLR for the period 2011-2100. Incremental rises in sea level at decadal time steps for this period were drawn from the A1FI 95% IPCC^4^ SLR scenario in the sourth assessment report (AR4). SEC at each time step was calculated utilising Equation 3, with the temporally-adjusted elevation layer developed for the previous time step being utilised as parameter *H* for the calculation of wetland surface elevation. DEM3 was utilised as the initial grid-based elevation surface.

For each decadal time increment to the year 2100, the wetland vegetation distributions were modelled based on the approximate maximum and minimum elevations of each wetland community type at the Minnamurra site. Wetland elevation boundaries that were previously defined^10^ were implemented in the classification process, further aiding in subsequent model comparability.

Comparison Model 2

An empirically based spatial model that simulates wetland surface elevation and vegetation distribution under conditions of rising sea levels has been developed for the study site^10^. The model is based upon a prior technique^11^ whereby significant factors to be modelled are identified in an initial stepwise regression, multiple potential equations are developed and a final model chosen that provides the best fit with site-specific accretion and surface elevation trend data. Supplementary Table 7 presents the models that best described accretion and elevation increments. Factors identified as significant and implemented in the models included time (i.e. days from first SET measurement), average rainfall for the previous month (RFI1), 6-month average water level (6MMWL), average annual rainfall (AAR), distance to the shore (DTS), 3-month averaged southern oscillation index value (SOI2) and the mean sea level (MWL).

Wetland evolution under SLR was conducted at decadal time increments from 2011 until 2100 using DEM3 in this study. Sea-level rise projections became the input variables for MMWL and MWL at each time step. As reliable projections of rainfall and SOI are not available to 2100, these were held constant and based on rates indicated in Supplementary Table 8. DTS was estimated at each time step and calculated as the distance of every cell to the shore, where shore is defined was the position of 0 m AHD in 2011 (Supplementary Table 8). Model simulation was undertaken using the ArcGIS Raster Calculator tool. Initially the accretion increment was determined to create an accretion increment surface. This accretion surface then became an input variable in the generation of an elevation increment surface, again using the raster calculator tool. The generated elevation increment surface was then added to DEM3 to create a new DEM at time step 1. This procedure was repeated; however the elevation increment was subsequently added to the prior generated DEM, rather than the initial DEM3.

Vegetation distribution was determined at each time step to the year 2100. The wetland vegetation distributions were modelled based on the approximate maximum and minimum elevation boundaries of each wetland community type at the Minnamurra site, as previously defined^10^.

Supplementary Table 7: The equations and coefficient of determination derived from factorial analysis that describe accretion and elevation increments for each time step in CM2.

| **Component** | **Equation** |
| --- | --- |
| Accretion (r^2^ = 0.73) | 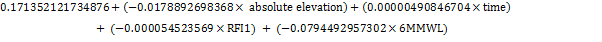 |
| Elevation (r^2^ = 0.68) | 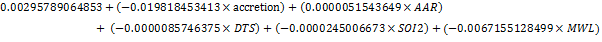 |

Supplementary Table 8: Values for variables used for the accretion and elevation model increments for each time step in CM2.

| **Variable** | **Values used when implementing model projections** |
| --- | --- |
| Time | in days since the first SET-MH measurement at the site |
| RFI1 | kept constant as the average value for April 2011 when the GPS survey was done |
| 6MMWL | increased according to the A1FI 95 % CI or B1 5 % CI IPCC sea-level rise scenario with a starting value of 1.9720 m—the Port Kembla mean maximum for the 6 months prior to April 2011 |
| AAR | kept constant as the average value from all weather stations used in the FA model |
| DTS | the calculated distance of every cell in the grid to the shore where shore is defined as the position of 0 m AHD sea level in 2011. For FA 1 this was constant through time. For FA 2 distance to shore was recalculated according to sea-level rise for each time increment |
| SOI2 | kept constant as the overall average SOI value |
| MWL | changing through time according the IPCC 95 % A1FI or the 5 % B1 sea-level scenario |

References

1 Temmerman, S., Govers, G., Wartel, S. & Meire, P. Spatial and temporal factors controlling short-term sedimentation in a salt and freshwater tidal marsh, Scheldt estuary, Belgium, SW Netherlands. *Earth Surface Processes and Landforms* **28**, 739-755, doi:10.1002/esp.495 (2003).

2 Cowell, P. & Thom, B. in *Coastal Evolution: Late Quaternary Shoreline Morphodynamics* (eds RWG Carter & CD Woodroffe) 33-86 (Cambridge University Press, Cambridge, United Kingdom and New York, NY, USA, 1994).

3 Wright, L. D. & Thom, B. G. Coastal depositional landforms: a morphodynamic approach. *Progress in Physical Geography* **1**, 412-459 (1977).

4 IPCC. Climate Change 2007: The Physical Science Basis. Contribution of Working Group I to the Fourth Assessment Report of the Intergovernmental Panel on Climate Change. (Cambridge University Press, 2007).

5 Allen, J. R. L. Simulation models of salt-marsh morphodynamics: some implications for high-intertidal sediment couplets related to sea-level change. *Sedimentary Geology* **113**, 211-223, doi:http://dx.doi.org/10.1016/S0037-0738(97)00101-2 (1997).

6 Allen, J. R. L. Salt-marsh growth and stratification: A numerical model with special reference to the Severn Estuary, southwest Britain. *Marine Geology* **95**, 77-96, doi:http://dx.doi.org/10.1016/0025-3227(90)90042-I (1990).

7 Allen, J. R. L. Salt-marsh growth and fluctuating sea level: implications of a simulation model for Flandrian coastal stratigraphy and peat-based sea-level curves. *Sedimentary Geology* **100**, 21-45 (1995).

8 French, J. R. Numerical simulation of vertical marsh growth and adjustment to accelerated sea-level rise North Norfolk, UK. *Earth Surface Processes and Landforms* **18**, 63-81 (1993).

9 MHL. OEH NSW Tidal Plane Analysis: 1990-2010 harmonic analysis. (2012).

10 Oliver, T., Rogers, K., Chafer, C. & Woodroffe, C. Measuring, mapping and modelling: an integrated approach to the management of mangrove and saltmarsh in the Minnamurra River estuary, southeast Australia. *Wetlands Ecology and Management* **20**, 353-371, doi:10.1007/s11273-012-9258-2 (2012).

11 Rogers, K., Saintilan, N. & Copeland, C. Modelling wetland surface elevation dynamics and its application to forecasting the effects of sea-level rise on estuarine wetlands. *Ecological Modelling* **244**, 148-157, doi:10.1016/j.ecolmodel.2012.06.014 (2012).
